# Optimization of Bioactive Ingredient Extraction from Chinese Herbal Medicine* Glycyrrhiza glabra*: A Comparative Study of Three Optimization Models

**DOI:** 10.1155/2018/6391414

**Published:** 2018-05-15

**Authors:** Li Yu, Weifeng Jin, Xiaohong Li, Yuyan Zhang

**Affiliations:** ^1^College of Life Science, Zhejiang Chinese Medical University, Hangzhou 310053, China; ^2^College of Pharmaceutical Science, Zhejiang Chinese Medical University, Hangzhou 310053, China

## Abstract

The ultraviolet spectrophotometric method is often used for determining the content of glycyrrhizic acid from Chinese herbal medicine* Glycyrrhiza glabra*. Based on the traditional single variable approach, four extraction parameters of ammonia concentration, ethanol concentration, circumfluence time, and liquid-solid ratio are adopted as the independent extraction variables. In the present work, central composite design of four factors and five levels is applied to design the extraction experiments. Subsequently, the prediction models of response surface methodology, artificial neural networks, and genetic algorithm-artificial neural networks are developed to analyze the obtained experimental data, while the genetic algorithm is utilized to find the optimal extraction parameters for the above well-established models. It is found that the optimization of extraction technology is presented as ammonia concentration 0.595%, ethanol concentration 58.45%, return time 2.5 h, and liquid-solid ratio 11.065 : 1. Under these conditions, the model predictive value is 381.24 mg, the experimental average value is 376.46 mg, and the expectation discrepancy is 4.78 mg. For the first time, a comparative study of these three approaches is conducted for the evaluation and optimization of the effects of the extraction independent variables. Furthermore, it is demonstrated that the combinational method of genetic algorithm and artificial neural networks provides a more reliable and more accurate strategy for design and optimization of glycyrrhizic acid extraction from* Glycyrrhiza glabra*.

## 1. Introduction

*Glycyrrhiza glabra* or* G. glabra*, one of the most widely used traditional Chinese medicines in China, has the effect of invigorating spleen and replenishing qi and clearing away heat and toxic substances to treat diseases like weakness of spleen and stomach, cough and phlegm, and so on [1]. Owing to its anti-inflammatory, antispasmodic, antiallergic, antidepressive, antiviral, antifungal, and antioxidant activities, it has drawn more and more attentions [2–5]. Particularly, the long-term clinical practice has demonstrated that it exhibits the functions of alleviating pain, tonifying spleen and stomach [6], eliminating phlegm [7], and relieving coughing [8], etc. Meanwhile, it is also a highly nutritional plant, which is widely used as an important sweetening and flavouring agent in food products, such as candies, chewing gum, toothpaste, and beverages [9, 10]. Recently, the in-depth studies have uncovered that there exist more than 400 isolated compounds and active ingredients from this herb [11].

Among these constituents, glycyrrhizic acid has been reported to be the main biologically active ingredient in* Glycyrrhiza glabra*, as it is thought to be responsible for the hepatic protective and antiulcer effects of* Glycyrrhiza glabra* [12, 13]. Meanwhile, this active substance has been widely studied for their pharmacologic effects and medical benefits in animal models and human studies [14, 15]. For example, a considerable interest has been attained to the glycyrrhizic acid for its critical pharmacological activities, including anti-inflammatory, antioxidative, and antitumor activities [16]. Henceforth, it is necessary to develop an accurate, precise, and reliable prediction method for analysis of glycyrrhizic acid from* Glycyrrhiza glabra*.

Based on the application of industrial extraction technologies, the extraction process has been greatly improved, and the corresponding cost has been reduced [17]. However, the analysis and interpretations of the optimum extraction conditions seem to be empirical, and the research of some parameters is still under serious investigations. To address these problems, the traditional single variable approach, in which the level of each parameter is varied individually, while those of the others hold constant, spends too much time and requires a large number of experiments [18]. Accordingly, the advanced multivariable methodologies for the analysis of optimal extraction process are highly demanded [19], with the purpose of obtaining high-quality active substance of glycyrrhizic acid. For example, the central composite design (CCD), firstly presented by Box and Wilson [20], is a typical one. This design consists of the following parts: a full factorial or fractional factorial design, an additional design, and a central point.

Various prediction models and methods have been proposed for the analysis of extraction process. These methods can be roughly grouped into two categories. The first class is based on the experimental analysis and is mostly relied on assumption for model simplification. Such an extraction process model is evaluated statistically [20, 21]. For example, response surface methodology (RSM) is the most relevant statistical technique used in the analytical optimization. RSM, which is a collection mathematical and statistical techniques, can be well applied for the fit of a polynomial equation to the experimental data with the objective of making statistical prevision. To understand and characterize the highly nonlinear relationships between the extraction and response variables, the second class is inspired by the data-driven techniques [22]. As an example, the artificial neural network (ANN) methodology is one of the most promising artificial intelligence methods [23, 24]. ANN analysis provides the modeling of complex relationships and is quite flexible in regard to the number and form of the experimental data. This makes it possible to use more informal experimental designs than with statistical approaches, potentially making the ANN model more accurate.

Over the years, the backward propagation neural network (BPNN), which is a typical ANN, has been widely employed to unveil the complicated relationships between the input and output variables [25]. Since the weights of BPNN are trained with an optimization method such as gradient descent algorithm, it is not possible to guarantee to find the global minimum solutions, but only a local minimum [26]. As can be seen, genetic algorithm (GA), which simulates the survival-of-the-fittest principle of nature, is commonly used in searching for global optimum in the entire solution space [27]. Besides, GA is applicable to solve a variety of optimization problems, specifically the discontinuous, no differentiable, stochastic, and highly nonlinear objective functions [28]. To overcome the limitation of local minimum performance of backward propagation algorithm, the optimization of weights of BPNN, therefore, can be implemented by GA. This combinational method, abbreviated as GA-BPNN, has been successfully applied in diverse fields [29–31].

Depending on the single variable approach, four extraction parameters of ammonia concentration, ethanol concentration, circumfluence time, and liquid-solid ratio, which strongly influence the content of glycyrrhizic acid, are chosen as the independent variables in the present study [32]. Furthermore, CCD of four factors and five levels is taken into consideration for the experiments of glycyrrhizic acid extraction from* Glycyrrhiza glabra*. After acquiring data related to each experimental point, the predictable models of RSM, BPNN, and GA-BPNN are developed to describe the interaction between the different experimental variables and approximate a nonlinear response function to experimental data for the extraction process of glycyrrhizic acid. According to the above well-established models, GA is assigned to search for optimizing the extraction parameters of glycyrrhizic acid. For the first time, a comparative study of these three computational models is conducted for the evaluation and optimization of the effects of the extraction independent variables on the extraction efficiency of glycyrrhizic acid from* Glycyrrhiza glabra*. Additionally, it is shown that GA-BPNN is more reliable and accurate and has better predictive power than two other models for optimization of glycyrrhizic acid extraction from* Glycyrrhiza glabra*.

## 2. Materials and Experimental Design

### 2.1. Reagents and Materials

**Figure 1:**
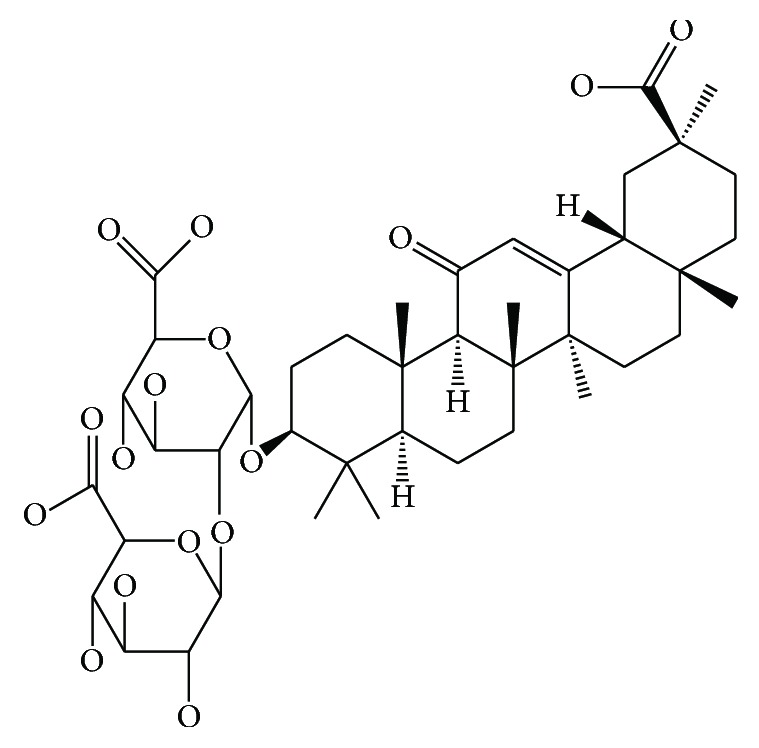
The chemical structure of glycyrrhizic acid.

The herbal drug—*Glycyrrhiza glabra* (batch number: 150701)—was purchased from Huqingyutang pharmacy (Zhejiang province, China) and was identified by Shenwu Huang, the professor of Zhejiang Chinese Medical University. The crude slices were of the stipulated quality standards in Chinese Pharmacopoeia (2015 edition). Glycyrrhizic acid (batch number: 110731-201517, purity: ⩾98%) was purchased from National Institute for the Control of Pharmaceutical and Biological Products (Beijing, China). The chemical structure of glycyrrhizic acid is shown in Figure 1. The other reagents were of analytical grade. The working solutions of glycyrrhizic acid were prepared by diluting appropriate amounts of the stock solutions with buffer solutions. The FA1004N analytical balance (Precision Instrument Co., Ltd., Shanghai, China) was utilized to accurately weigh the materials. The 018268 type electric-heated thermostatic water bath (Automation Instrument Factory, Suzhou, China) was used to extract the glycyrrhizic acid. The TU 1900 type double-beam UV-visible spectrophotometer (Puxi General Instrument Co., Ltd., Beijing, China) was prepared to detect the content of glycyrrhizic acid. Finally, the Milli-Q (Millipore, Bedford, MA, USA) purification system was employed to provide the deionized water for preparing all the required solutions.

### 2.2. Calibration Curve and Methodological Study

Here, the methodological study was in line with [32]. For preparation for sample solutions, 5.00 g powdered drug of* Glycyrrhiza glabra* was precisely weighed and extracted by the conditions as listed in (1). The method of condensing and heat reflux with ammonia-ethanol was used in this paper. Then, the mixture was filtered and transferred quantitatively to a 100 ml measuring flask with ethyl alcohol. After beinf diluted to 100 ml, the solutions were processed through 0.22 *μ*m syringe filter and subsequently detected under the established ultraviolet conditions.

(1) 0.5%≤ammonia  concentration  A≤0.7%,50%≤ethanol  concentration  B≤70%,1.5 h≤circum  fluence  time  C≤2.5 h,10:1≤liquid-solid  ratio  D≤12:1.

To verify the reliability of experimental methodology, the following four aspects were proposed as the evaluation criteria:

- *Calibration curve*: seven working solutions, whose respective concentrations were as follows, 8, 16, 24, 32, 40, 48, and 56 *μ*g/ml, were exploited to make the calibration curve of glycyrrhizic acid, where the value of optical density (*Y*) and its corresponding standard concentration (*X*) matched a linear regression curve. Subsequently, one of the glycyrrhizic acid working solutions was scanned in the whole wavelength, and the maximum absorption wavelength of glycyrrhizic acid was detected at 252 nm. Consequently, the linear regression of glycyrrhizic acid for the calibration curve was calculated as *Y* = 12.674*X* + 0.0303 with the fitting degree 0.9995 and the concentration range 8 *μ*g/ml–56 *μ*g/ml.
- *Precision*: for this part, the within-day and between-day precision was checked. To determine the within-day precision, one working solution (56 *μ*g/ml) was examined 5 times in the same day. To determine the between-day precision, the same working solution was analyzed on other 5 consecutive days. The relative standard deviation (RSD) was taken as a metric of precision. The fact that RSD of within-day and between-day precision were 0.17% and 0.65%, respectively, implied that the developed UV detection method was feasible.
- *Stability*: the stability of sample solutions was measured after 0 h, 2 h, 4 h, 6 h, and 8 h at room temperature under the selected UV detection conditions. Similarly, RSD was taken as an evaluation metric. Through the experimental tests, the stability trend of 5 samples fluctuated up and down which was no significant difference. And the RSD was 0.884%, which suggested the stability of sample solutions at room temperature.
- *Recovery*: to evaluate the property of recovery, three working solutions (16, 32, and 48 *μ*g/ml) were used in recovery test. The absorbance values were taken into the calibration curve. The corresponding results of recovery for 16, 32, and 48 *μ*g/ml (expressed by “mean value ± standard deviation” with *n* = 6) were 98.032% ± 6.031%101.026% ± 3.094%, and 99.877% ± 1.739% respectively.

### 2.3. Experimental Design and Data Normalization

Before applying the predictable models, it is necessary to choose an experimental design to define which experiments should be carried out in the experimental region being studied. In this work, the independent variables with major effects on the extraction process to determine glycyrrhizic acid from* Glycyrrhiza glabra* were selected through the single variable approach. These parameters and their delimitation for the extraction experiments are displayed in (1).

In order to evaluate the coefficients of interaction parameters, CCD was identified to carry out the experiments. The domain of variation for each factor was determined based on knowledge of the system and acquired from initial experimental trials. Their ranges and levels with actual and coded values of each parameters were shown in Table 1, where the independent variables were coded to two levels, namely, low (−1) and high (+1), whereas the axial points were coded as −2 and +2. Then, the Design-Expert software (version 8.01) was used for this experimental design matrix. Totally, 30 experimental points, including 16 factorial points, 8 axial points, and 6 replicated at the center points, were defined with four independent factors and five levels. All the runs were conducted in duplicate randomly to minimize the experimental errors, as well as to verify the adequacy of the proposed models. Eventually, the complete CCD matrix in terms of coded variables *x*_*i*_  (*i* = 1,2, 3,4), as well as experimental results, is exhibited in Table 2.

Codification of the levels for each variable consists of transforming the studied real values into coordinates inside a scale with dimensionless values, which must be proportional at their localization in the experimental space. Moreover, it cancels the order of magnitude difference between the extraction parameters and avoids causing large prediction error. The results improve the learning efficiency and the prediction accuracy of models. Therefore, one can use this codification schematic as the normalization process for the experimental data. Precisely, the normalization process is applied to transform a real value (*z*_*i*_) into a coded value (*x*_*i*_) according to the following equation:

(2) $$\begin{array}{c} x_{i}=\frac{z_{i}-z_{i}^{0}}{\Delta z_{i}}, \end{array}$$

where *x*_*i*_ is the dimensionless value of the independent variable *i*, *z*_*i*_ and *z*_*i*_^0^ is actual value and that at the central point, respectively, and Δ*z*_*i*_ is the step change of *z*_*i*_ corresponding to a unit variation of the dimensionless value. The related parameters for each independent variable normalization are also displayed in Table 1.

## 3. Models and Optimization

### 3.1. Determination of the Optimal Extraction Parameters

GA is a parameter searching and optimization technique based on emulation of nature evolutionary processes. In a GA, a population of candidate solutions (also called individuals) to an optimization problem is evolved toward the best solution. Individuals are represented in binary as strings of 0*s* and 1*s*, but other encodings are also possible. In each generation, the fitness, which is usually the value of the objective function in the optimization problem being solved, is assessed for each individual in the population. The more fit individuals are stochastically selected from the current population, and the next new population of candidate ones is created through the bio-inspired operators, such as selection, mutation, and crossover. As the algorithm proceeds, the best fitness of the population is gradually improved. Commonly, the algorithm terminates when either a maximum number of generations has been achieved or a satisfactory fitness level has been reached.

In light of the powerful search function, GA was exploited to optimize the extraction conditions for glycyrrhizic acid throughout this work. Each individual was represented by the extraction parameters *A*, *B*, *C*, and *D*, with values within the variable upper and lower bounds as defined in (1). The fitness functions were identified by the well-constructed mathematical models, which are presented in the following subsections.

### 3.2. RSM and Statistical Analysis

In statistics, CCD is the most popular class of design used for fitting a second-order model in RSM, especially in the extraction process. Based on the obtained experimental data, RSM with a second-degree polynomial formula was applied to explore the relationship between four explanatory variables of *A*, *B*, *C*, and *D* and one response variable of glycyrrhizic acid, which can be seen in the following:

(3) $$\begin{array}{c} y=a_{0}+\overset{4}{\underset{i=1}{\sum }}a_{i}x_{i}+\overset{4}{\underset{i=1}{\sum }}\overset{4}{\underset{j=1}{\sum }}a_{ij}x_{i}x_{j}+\varepsilon , \end{array}$$

where *y* expresses the content of glycyrrhizic acid, *x*_*i*_ (*i* = 1,2, 3,4) represent the extraction parameters, *a*_0_ is the constant term, *a*_*i*_ (*i* = 1,2, 3,4) are the coefficients of the linear part, *a*_*ij*_ (*i*, *j* = 1,2, 3,4) indicate the coefficients of the quadratic part, and *ε* means the residual associated with the experiments.

The mathematical model, found after fitting the function to the data, can sometimes not satisfactorily describe the experimental domain studied. Based on the multiple sample mean data, the more reliable way to evaluate the quality of the fitted model is by the application of one-way analysis of variance (ANOVA). In this work, the data were analyzed by the Design-Expert software (version 8.01), and the coefficients were interpreted by Fisher's test. The statistically nonsignificant terms were omitted in the specific model. The accuracy and general ability of the polynomial model fitted can be evaluated by the coefficient of determination *R*^2^, which is defined as follows:

(4) $$\begin{array}{c} R^{2}=1-\frac{\sum_{i}{\left(y_{i,p}-y_{i,e}\right)}^{2}}{\sum_{i}{\left(y_{i,e}-\overset{-}{y}\right)}^{2}}, \end{array}$$

where *y*_*i*,*p*_ is the value predicted by the model, *y*_*i*,*e*_ is the experimental value, and $\overset{-}{y}$ is the mean of experimental values. It is worth mentioning that *R*^2^ is only applicable to the training data set, and its range is [0,1]. In addition, the larger *R*^2^ indicates that the more percent of the variance in the response variable can be explained by the explanatory variables.

### 3.3. BPNN Model

**Figure 2:**
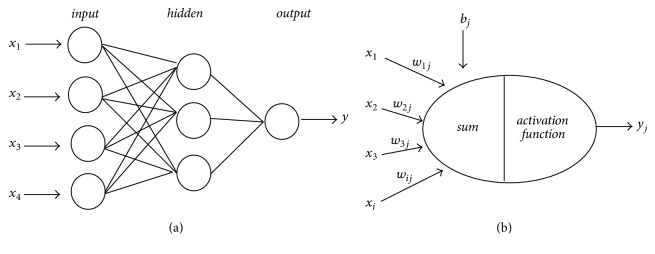
Artificial neural network: (a) general schematic of a three-layer network; (b) operation of a single neuron through an activation function.

ANN is a novel information processing technique and a simplified computational model, which is enlightened by the structure of biological neural networks. It often consists of three layers, i.e., input, hidden, and output layers. The pattern of interconnection among the neurons is called the network structure, and it can be conveniently illustrated by a graph as shown in Figure 2(a). Data generated from the experimental design can be used as relevant inputs and outputs for ANN training.

The training is carried out by adjusting the strength of connections between neurons with the aim of adapting the outputs of the entire network to be closer to the desired outputs. In this approach, sum of inputs arrived at each neuron is weighted, and an output signal is generated through an activation function as

(5) $$\begin{array}{c} y_{j}=f\left(\;\overset{n}{\underset{i=1}{\sum }}\omega_{ij}x_{i}+b_{j}\;\right), \end{array}$$

where *ω*_*ij*_ and *b*_*j*_ (*i*, *j* = 1,2,…, *n*) are the weights between two sequential layers and *x*_*i*_ and *y*_*i*_ (*i* = 1,2,…, *n*) are the corresponding inputs and outputs, respectively. A general schematic of such architecture is illustrated in Figure 2(b).

Then, the network calculates the output values and obtains the evaluation criteria by comparing the predicted values and the experimental values. After that, the network updates the weights to improve the criteria and achieves the optimal goals through a neural network learning algorithm. In the present work, a three-layer BPNN was developed for explaining the extraction mechanism of glycyrrhizic acid, in the sense that the weights were updated via the backward propagation algorithm.

The performance of the model is statistically evaluated by the following two evaluation criteria: the maximum absolute error MAE and the correlation coefficient *r*, as well as the coefficient of determination *R*^2^. The former two matrices are calculated as follows:

(6) MAE=maxi yi,p−yi,e,r=∑iyi,p−y−i,pyi,e−y−i,e∑iyi,p−y−i,p2∑iyi,e−y−i,e2,

where $y_{i,p},\;{\overset{-}{y}}_{i,p}\;\;(i=1,2,\ldots ,n)$ are the predicted values and the corresponding mean values, respectively, and, $y_{i,e},\;{\overset{-}{y}}_{i,e}\;\;(i=1,2,\ldots ,n)$ are the experimental values and the corresponding mean values, respectively. In particular, the evaluation criterion MAE is stricter than those metrics of mean absolute error and root mean square error, etc.

In order to avoid the overlearning of the model data, the number of hidden nodes was increased from *n*_1_ to *n*_2_, and a *k*-fold cross-validation method was applied in this work, where all data were randomly subdivided into two distinct groups: the training set was used to train the network, and the testing set was used to evaluate its performance.

### 3.4. GA-BPNN Method

**Figure 3:**
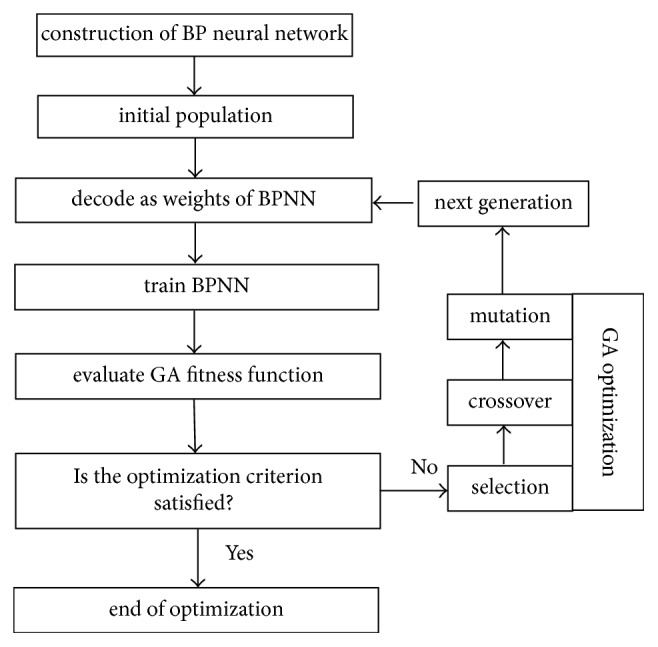
Schematic of optimization procedure of GA-BPNN.

Although the backward propagation method is the best known example of neural network learning algorithm, it has trouble crossing plateaux in the error function landscape. This issue results in the drawback of the local optimum to calculate the gradient of the loss function with respect to the weights of networks. In this study, GA was applied to optimize the parameters of BPNN, and the outline of the combination GA-BPNN model is depicted in Figure 3. As can be seen in Figure 3, the new combinational algorithm can be formalized by the following two parts:

- *Determining BPNN structure*: the input layer consists of four nodes related to the independent extraction variables and the output layer has one node associated with the response of glycyrrhizic acid. The optimal number of hidden nodes is determined among examined neurons from *n*_1_ to *n*_2_.
- *Utilizing GA to optimize BPNN*: a population with *N* individuals is generated randomly, and the corresponding individuals are decoded into the network weights. Then, BP algorithm is employed to update the weights, and the fitness value of each individual is assessed. Finally, the best individual is found by selection, crossover, and mutation operators, and the corresponding BPNN model is confirmed.

The performance of BPNN was measured by the mean square error attached to Matlab 2015a. Meanwhile, a *k*-fold cross-validation method was also applied. In the iterative optimization, the weights were encoded by real encoding, and the encoding length could be calculated by the equation as *S* = *RS*_1_ + *S*_1_*S*_2_ + *S*_1_ + *S*_2_, where *R*, *S*_1_, and *S*_2_ are the number of input, hidden, and output nodes, respectively. The tangent sigmoid transfer function at both hidden and output layers was successfully employed. Network training was performed by *M* epochs. The fitness function of each individual was defined as the maximum value between the maximum absolute error MAE_train_ of the training set and the maximum absolute error MAE_test_ of the testing set, in view of the following:

(7) fitness=max MAEtrain,MAEtest.

## 4. Results and Discussion

Experimental results for optimizing four factors according to the selected CCD are shown in Table 2. The average value of runs 6, 18, 21, 22, 28, and 30, carried out at the central point, was 365.45 mg, which indicated that the extraction ability of glycyrrhizic acid was stronger by comparing to the other runs in this experimentation. Meanwhile, the relative standard deviation was 0.46%, which showed that the experiments were stable. The highest value (376.46 mg) was obtained in run 12 with the extraction conditions as *x*_1_ = 1, *x*_2_ = 1, *x*_3_ = −1, and *x*_4_ = 1 and thereby highlighted the importance of the changes of these conditions to enhance extraction yield. The lowest value (290.93 mg) was marked in run 7 with the extraction conditions as *x*_1_ = −1, *x*_2_ = 1, *x*_3_ = −1, and *x*_4_ = −1. By comparison with the extraction parameters of these two extreme values, the conditions of ammonia concentration and liquid-solid ratio might exert significant effects on the response *y*.

To disclose the more precise relationships between the independent and dependent variables, the previously introduced RSM, BPNN, and GA-BPNN models in combination with experimental design were utilized to optimize the extraction conditions for glycyrrhizic acid from* Glycyrrhiza glabra*. The corresponding results and analysis are presented in the following subsections.

### 4.1. Modeling and Optimization by RSM

From the regression analysis applied to the results in Table 1, the following model of RSM of (8) is derived for the content of glycyrrhizic acid (*y*) as function of the extraction conditions *x*_1_, *x*_2_, *x*_3_, and *x*_4_, where the coefficients are estimated via the least squares method, and the statistically nonsignificant ones (*p* > 0.05) are removed.

(8) y=365.45+11.92x1+11.95x3+11.45x4−14.1x3x4−8.25x12−9.68x22.

The corresponding ANOVA results are displayed in Table 3.

According to (8), the negative coefficients for the model terms *x*_3_*x*_4_, *x*_1_^2^, and *x*_2_^2^ indicate the unfavorable effects on the extraction of glycyrrhizic acid; the positive coefficients for the model terms *x*_1_, *x*_3_, and *x*_4_ mean the favorable effects on the dependent variable. Meanwhile, the goodness of fit of regression equation can be assessed by adjusted determination coefficient of *R*^2^. The values *R*^2^ of 0.9465 and adjusted *R*^2^ of 0.8966 show that the model could be significant predicting the response and explaining approximately 90% of the variability in the extraction of glycyrrhizic acid. Generally, the model *F*-value of 18.96 implies that the model is significant and shows that the model is statistically significant at 95% confidence level (*p* < 0.0001).

**Figure 4:**
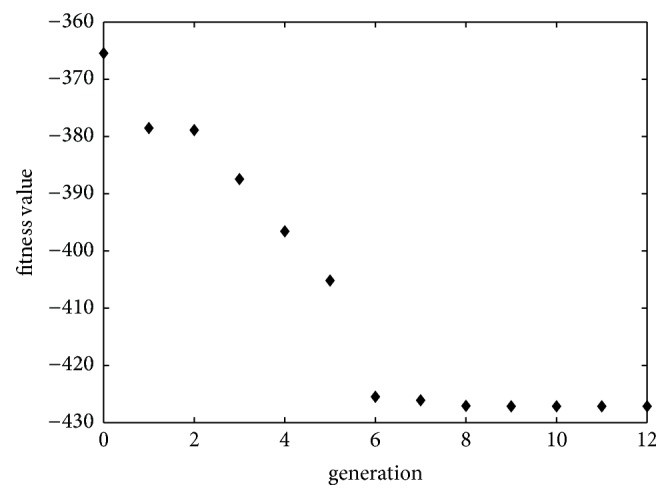
Plot of best fitness by GA method. The fitness function is defined in equation (4), and the maximum content of glycyrrhizic acid is read as 427.156 mg with the optimization extraction conditions: 0.722, 0, 2, and −2 in the coded form.

Despite the nonsignificant coefficient of the linear term *x*_2_, the parameter *x*_2_ still negatively influences the response value by virtue of its significant coefficient of quadratic term. According to ANOVA, pred-*R*^2^ value is 0.6954, and the lack of fit is statistically significant, which both reflects that this model is invalidated for predictive purpose. Indeed, the profile for predicted values and desirability option in the GA toolbox of Matlab 2015a was used for the optimization process. Each individual was represented by the extraction parameters *x*_1_, *x*_2_, *x*_3_, and *x*_4_, with values within the variable upper and lower bounds in Eq. (1), and the fitness function was the regression equation Eq. (8). In addition, GA was processed with 12 generations (Figure 4), population size of 30, and the rest setting as default. As a result, the maximum content of glycyrrhizic acid (427.1562 mg) was predicted by GA at the following conditions: 0.722, 0, 2, and −2 in the coded form (Table 4). Under these extraction conditions taken into account, the experimental results (Table 5) were conducted. Therefore, it could be concluded that this model cannot be considered a good choice for modeling the experimental data of this study. Nonetheless, it is presented here only for comparison with BPNN and GA-BPNN modeling.

### 4.2. Modeling and Optimization by BPNN

Firstly, the 10-fold cross-validation method was adopted to divide the input data into two distinct sets: the training set of 90% input data and the testing set of 10% input data. Meanwhile, BPNN toolbox of Matlab 2015a with the maximum epochs 2000 was applied for BPNN model. The relation among the number of hidden neurons, *R*^2^, *r*, and MAE is shown in Table 6.

As can be seen in Table 6, MAE for the training set has a significant improvement in performance by increasing the number of hidden layer, whereas, for the testing set, its performance exhibits the negative effect for 5, 7, 8, and 9 neurons, respectively. Combined with the correlation coefficient *r*, the optimal structure of the network with 4 neurons in the hidden layer is applied for further prediction.

**Figure 5:**
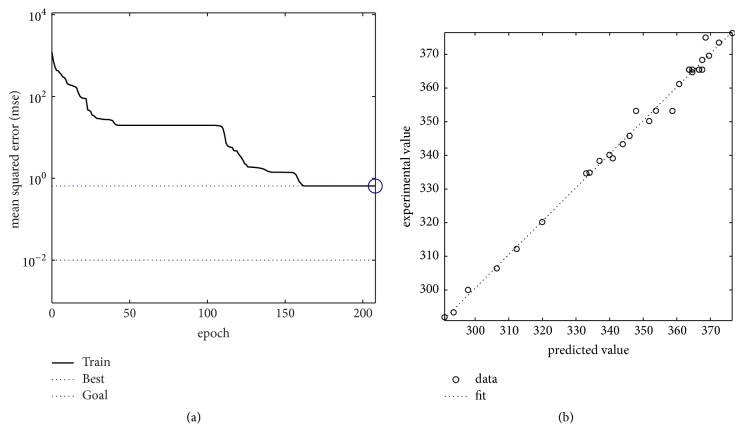
(a) Learning performance curve for the whole data, and the best training performance is 0.6493 at epoch 208. (b) Experimental and BPNN predicted results for glycyrrhizic acid for the whole data. The number of hidden neurons of BPNN is 4, and the correlation coefficient is *r* = 0.9975.

Thereafter, the whole data were used to train the neural network. The learning curve for training is given in Figure 5(a). As can be found in Figure 5(a), mean square error decreases initially and then it becomes almost constant. Moreover, the trained network is used to estimate the response of 30 experimental points, and the correlation coefficient between actual and estimated responses is *r* = 0.9975, as shown in Figure 5(b).

After being well trained, an optimization was then performed using GA, whose results are also listed in Table 4. As already mentioned, this model was obtained so as to deliberately overtrain the network. Despite the better performance obtained for this model, it cannot be considered a good choice being obtained through an inadequate training/testing methodology. Hence, there was a bad agreement between the BPNN predictions and experimental data (Table 5) with the above optimum conditions. Although the optimization cannot be considered reliable due to the above explanation, it is presented here only for comparison with RSM and GA-BPNN modeling.

### 4.3. Modeling and Optimization by GA-BPNN

As already mentioned, BPNN is an effective data processing method. But the problem is that BP algorithm is easy to get stuck in local minimum in the sense that the different original weights always give rise to the different training epochs. In this regard, GA was combined with BPNN to optimize the initial distribution of weights and enable BPNN to fit not only the training data, but also the testing data very well.

**Figure 6:**
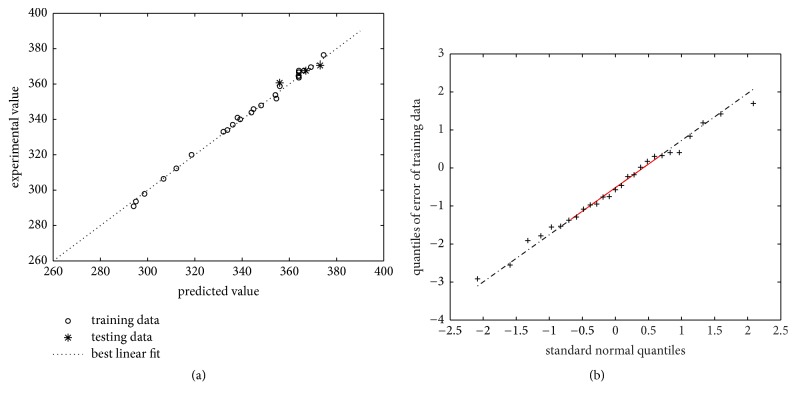
(a) Experimental and GA-BPNN predicted values of glycyrrhizic acid for the training and testing data. The number of hidden neurons of BPNN is seven. (b) Q-Q plot of standard normal distribution versus error data of the training set.

Similarly, GA toolbox of Matlab 2015a was applied for GA-BPNN model. The relationships among the number of hidden neurons, *R*^2^, *r*, and MAE are shown in Table 7. The main criterion for selection of the optimum BPNN structure is the MAE of the test data as well as the correlation coefficient *r*. As can be found in Table 7, the values of *r* and MAE both reach the best performance if there are 7 hidden neurons. Moreover, the corresponding weights of BPNN are calculated as listed in Table 8. The plot between the measured and model-predicted values is illustrated in Figure 6(a), which implies that BPNN model with 7 hidden neurons is consistent with the experimental data. It should be noted that the Q-Q probability plot of the prediction residuals can provide additional information regarding model fitting to a data set. In fact, a careful examination of Q-Q plot in Figure 6(b) reveals that the probability distribution of residuals corresponds with the expected normal distribution of the test line, and it is demonstrated that the prediction performance of this model for glycyrrhizic acid with high confidence levels is credible. Henceforth, based on these considerations as a whole, one can infer that GA-BPNN model is the best modeling and optimization tool under the specific conditions selected for this work.

After modeling, an optimization was then performed using GA, whose results are listed in Table 4. The fitness function was the equation of BPNN model with the weights presented in Table 8. Compared with the experimental data in Table 5, it was truly remarkable that GA-BPNN model produced the best agreement between the predicted and the experimental values among these three models. The generalization ability of GA-BPNN is better; this may be due to that GA is good at global searching, and the weight adjustment is exquisite.

### 4.4. Comparative Study of RSM, BPNN, and GA-BPNN

On the one hand, Chinese herbology is the theory of traditional Chinese herbal therapy, which accounts for the majority of treatments in traditional Chinese medicine. There are roughly 13,000 medicinal plants used in China and over 100,000 medicinal recipes recorded in the ancient literature. Chinese herbal extracts are herbal decoctions that have been condensed into a granular or powdered form. For example, glycyrrhizic acid is the major active ingredient of Chinese herbal medicine* Glycyrrhiza glabra*, which has many pharmacological activities. On the other hand, RSM, BPNN, and GA-BPNN are three alternatively computational and predictable models capable of solving linear and nonlinear multivariate problems. In the present work, these three models were developed for describing the experimental data of the extraction of glycyrrhizic acid from* Glycyrrhiza glabra*. As a consequence, all models could be well fitted to the experimental response of glycyrrhizic acid. After being well established, GA was set to optimize the extraction conditions, which are summarized in Table 4 for the selected models. In order to further evaluate their accurate prediction and practicability, the extraction experiments were carried out for each of the predicted optimum conditions, and the corresponding results are displayed in Table 5. The existence of the high degree of agreement between the experimental results and predicted optimum results indicated that the GA-BPNN could be used effectively for the evaluation and optimization of the effects of the extraction independent variables on the extraction concentration of glycyrrhizic acid from* Glycyrrhiza glabra*.

It was indeed that RSM had a regression equation for forecasting and achieving optimum conditions for extraction process. However, classical RSM requires the specification of a polynomial function such as linear, first-order interaction, or second-order quadratic, to be regressed. Moreover, the number of terms in the polynomial is limited to the number of experimental design points. Hence, it has its drawbacks in providing the complex intrinsic relationships among input/output data set clearly. Also, this might be why it was less effective for predictive purpose and therefore was not suitable for the experimental data in this work. Although BPNN methodology provides the modeling of complex relationships, especially nonlinear ones, that may be investigated without complicated equations, it cannot be considered a good choice being obtained through an inadequate training/testing methodology.

Since GA has good global searching ability and can learn the near-optimum solution without the gradient information of error functions, it has been a powerful tool of optimization, searching, and machine learning. Additionally, the cross-validation method prevents the so-called overtraining responsible for a reduction of neural network ability to generalize knowledge. Comparing the discrepancy between the experimental and predicted data in Table 5, it is evident that GA-BPNN can be accepted as the most precise method within RSM and BPNN for modeling of the extraction of glycyrrhizic acid from* Glycyrrhiza glabra*.

As discussed in [29], GA-BPNN is not suitable for some complicated data sets. When data sets are complex, GA is so slow and hard to process them; it can only be treated as a presearch technique, that is, to find a better search space. However, GA-BPNN is not always valid; its parameters are also hard to decide. The future goals of this study include (1) applying this method for optimization of bioactive ingredient extraction from other Chinese herbal drugs; (2) adjusting related parameters to further improve the algorithm's efficiency.

### 4.5. Remarks

In the current studies of traditional Chinese medicine, a variety of data (such as extraction data, pharmacokinetics and pharmacodynamics data, and clinical data) are produced. The corresponding relationships are complex, and some are even random and fuzzy. Therefore, the deterministic approaches are often powerless, and one needs to appeal the new technologies, such as complex system and artificial intelligent. Particularly, machine learning, a fundamental concept of artificial intelligent research, has been demonstrated to possess the ability to describe the complex relationship between inputs and outputs. Moreover, the recently developed deep neural network has more powerful self-learning ability [26]. Indeed, the neural network technology in machine learning and genetic algorithm in global optimization algorithm have received extensive attention and extensive research and have shown an attractive application prospect.

It is remarkable that the evaluation and optimization of the extraction process of saponins and total flavonoids from* Glycyrrhiza glabra* have been conducted in [32]. Undoubtedly, it is a multilevel optimization problem, and the entropy weight method is used to assign the corresponding weights. Besides, the model of BPNN is developed for explaining the extraction mechanism. Through this previous study, it is found that BP algorithm is easy to get stuck in local minimum, and it means one should try a couple of times to obtain a satisfied result. In view of this fact, a combinational model of GA-BPNN is offered to be an alternative to RSM and BPNN as a modeling tool. Therefore, one component from* Glycyrrhiza glabra* is reconsidered as a single object optimization to illustrate the feasibility of the methodology proposed in this work. Frankly, the relevant results are incomparable, and the multilevel optimization problems are all important topics for further research in the near future.

## 5. Conclusion

In this study, the bioactive ingredient glycyrrhizic acid was successfully extracted from* Glycyrrhiza glabra*. By using central composite design, the time of analysis and experiment expense were decreased without obvious reduction in efficiency. Afterwards, the significant variables were optimized by RSM, BPNN, and GA-BPNN. These three models were compared for their predictive and generalization capabilities as well as their ability to optimize the concentration of glycyrrhizic acid. Comparing the results of numeric optimization and experiments through the above methods, it was shown that GA-BPNN was absolutely satisfactory owing to its adequate training/testing method. Under the reliable optimum conditions, the experimental and predicted content of glycyrrhizic acid were 379.46 mg, and 381.24 mg, respectively. Although GA-BPNN model has been successfully validated by the comparative results with two well-known correlations, it has its drawbacks in processing the more complex data sets. Nevertheless, the main benefits of GA-BPNN for extraction and determination of glycyrrhizic acid from* Glycyrrhiza glabra* are low sample consumption, minimum use of raw materials, simplicity, and high enrichment product.

## Figures and Tables

**Table 1:** Effective factors and levels for glycyrrhizic acid extraction from *Glycyrrhiza glabra*.

Coded levels	Ammonia concentration (%) (*A*)	Ethanol concentration (%) (*B*)	Return time (h) (*C*)	Liquid-solid ratio (*D*)
2	0.70	70	2.50	12.0 : 1
1	0.65	65	2.25	11.5 : 1
0	0.60	60	2.00	11.0 : 1
−1	0.55	55	1.75	10.5 : 1
−2	0.50	50	1.50	10.0 : 1
*z*_*i*_^0^	*z*_1_^0^ = 0.60	*z*_2_^0^= 60	*z*_3_^0^= 2.00	*z*_4_^0^ = 11.0 : 1
Δ*z*_*i*_	Δ*z*_1_ = 0.05	Δ*z*_2_ = 5	Δ*z*_3_ = 0.25	Δ*z*_4_ = 0.05

*Note.* For the independent variable *i*, *z*_*i*_^0^ is actual value at the central point and Δ*z*_*i*_ is the step change of the independent variable corresponding to a unit variation of the dimensionless value.

**Table 2:** Central composite design matrix and the experimental results for glycyrrhizic acid extraction from *Glycyrrhiza glabra*.

Run	*A* (*x*_1_)	*B* (*x*_2_)	*C* (*x*_3_)	*D* (*x*_4_)	Content of glycyrrhizic acid (mg)
1	1	−1	−1	1	360.68
2	0	0	2	0	372.52
3	1	1	1	−1	368.57
4	0	0	−2	0	333.06
5	1	1	1	1	353.78
6	0	0	0	0	366.60
7	−1	1	−1	−1	290.93
8	1	−1	−1	−1	312.35
9	−1	1	1	1	347.86
10	−1	−1	−1	1	343.91
11	−2	0	0	0	297.87
12	1	1	−1	1	376.46
13	0	2	0	0	334.03
14	−1	1	−1	1	339.97
15	0	0	0	2	369.56
16	−1	1	1	−1	345.89
17	1	1	−1	−1	306.43
18	0	0	0	0	366.60
19	1	−1	1	−1	364.62
20	1	−1	1	1	358.71
21	0	0	0	0	364.62
22	0	0	0	0	367.58
23	−1	−1	1	−1	340.95
24	−1	−1	−1	−1	293.61
25	2	0	0	0	367.58
26	0	0	0	−2	337.01
27	−1	−1	1	1	351.80
28	0	0	0	0	363.64
29	0	−2	0	0	319.94
30	0	0	0	0	363.64

*Note. x*_*i*_ is the dimensionless value of the independent variable *i*.

**Table 3:** Analysis of variance (ANOVA) for the fitted quadratic polynomial model for optimization of glycyrrhizic acid.

Score	Sums of squares	Degree of freedom	Mean squares	*F*-value	*p* value (Prob > *F*)
Model	17222.01	14	1230.14	18.96	<0.0001
*x*_1_	3410.55	1	3410.55	52.58	<0.0001
*x*_2_	41.19	1	41.19	0.63	0.4038
*x*_3_	3426.30	1	3426.30	52.82	<0.0001
*x*_4_	3149.21	1	3149.21	48.55	<0.0001
*x*_1_ *x*_2_	13.14	1	13.14	0.20	0.6591
*x*_1_ *x*_3_	50.13	1	50.13	0.77	0.3932
*x*_1_ *x*_4_	13.14	1	13.14	0.2	0.6591
*x*_2_ *x*_3_	0.65	1	0.65	0.000	0.9217
*x*_2_ *x*_4_	0.45	1	0.45	0.007	0.9348
*x*_3_ *x*_4_	3180.40	1	3180.40	49.03	<0.0001
*x*_1_^2^	1866.57	1	1866.57	28.77	<0.0001
*x*_2_^2^	2572.45	1	2572.45	39.66	<0.0001
*x*_2_^3^	286.71	1	286.71	4.42	0.0528
*x*_2_^4^	265.19	1	265.19	4.09	0.0614
Residual	973.04	15	64.87		
Lack of Fit	958.61	10	95.86	33.23	0.0006

	*R* ^2^	Adj-*R*^2^	Pred-*R*^2^	Adeq Precision	
	0.9465	0.8966	0.6954	15.413	

*Note*. *x*_*i*_ is the dimensionless value of the independent variable *i*, and *p* value < 0.05 is statistically significant.

**Table 4:** Optimized conditions in the coded form obtained by three different optimization models.

Optimization method	Ammonia concentration (%)	Ethanol concentration (%)	Return time (h)	Liquid-solid ratio
RSM	0.72	0	2	−2
BPNN	1.54	1.09	−1.23	1.38
GA-BPNN	−0.10	−0.31	2	0.13

**Table 5:** Experimental and predicted data of the content of glycyrrhizic acid from *Glycyrrhiza glabra* under the extraction conditions optimized by different models.

Optimization method	Number of experiments	Experimental value (mg)	Predicted value (mg)	Expectation discrepancy
RSM	3	369.23	427.1562	57.9262
BPNN	3	365.58	376.4012	10.8212
GA-BPNN	3	376.46	381.24	4.78

**Table 6:** Effect of the number of hidden neurons of BPNN on the maximum absolute error, the determination coefficient, and the correlation coefficient for the training data and the testing data.

Hidden neurons	MAE_train_	MAE_test_	*R*_trian_^2^	*r*_train_	*r*_test_
1	29.5138	12.3344	0.7102	0.7102	0.6638
2	24.6360	7.4841	0.8532	0.9237	0.9464
3	6.9000	21.7254	0.9844	0.9924	0.9889
4	6.6441	14.0391	0.9905	0.9952	0.9448
5	5.0962	40.3892	0.9935	0.9968	−0.1862
6	4.7070	44.1666	0.9921	0.9963	0.7794
7	6.0375	23.0501	0.9923	0.9964	−0.5058
8	2.4151	16.9665	0.9985	0.9963	−0.9959
9	5.2294	14.8987	0.9953	0.9980	−0.9267

*Note. *MAE_train_ and MAE_test_ are the maximum absolute error of the training set and the testing set, respectively. *R*_trian_^2^ is the determination coefficient of the training set. *r*_train_ and *r*_test_ are the correlation coefficient for the training data and the testing data, respectively.

**Table 7:** Effect of the number of hidden neurons of GA-BPNN on the maximum absolute error, the determination coefficient, and the correlation coefficient for the training data and the testing data.

Hidden neurons	MAE_train_	MAE_test_	*R*_train_^2^	*r*_train_	*r*_test_
1	24.8167	13.6733	0.6818	0.8260	0.5455
2	12.4153	11.4872	0.8422	0.9186	0.5325
3	6.9400	9.4277	0.9895	0.9952	0.5841
4	7.1209	6.8005	0.9857	0.9929	0.5667
5	4.1890	4.2232	0.9967	0.9984	0.7681
6	2.0200	4.8036	0.9984	0.9992	0.7052
7	3.8732	2.3678	0.9987	0.9979	0.9964
8	3.0094	3.2231	0.9967	0.9984	0.9957
9	6.8777	4.1785	0.9955	0.9979	0.9155

*Note. *MAE_train_ and MAE_test_ are the maximum absolute error of the training set and the testing set, respectively. *R*_train_^2^ is the determination coefficient of the training set. *r*_train_ and *r*_test_ are the correlation coefficient for the training data and the testing data, respectively.

**Table 8:** The weights of well-trained GA-BPNN with 7 hidden neurons for predicting the content of glycyrrhizic acid.

*W*_1_				*W*_2_	*B*_1_	*B*_2_
−1.7112	6.3033	2.1697	−1.9326	−0.7868	−1.6870	0.7532
0.6937	0.6410	0.6825	−6.2043	0.4207	28.0577	/
−0.2540	3.1229	−0.8732	22.4836	0.4419	−0.4640	/
4.1697	−0.6174	0.8745	3.9847	0.6316	0.5918	/
1.5632	0.4164	1.0697	5.9332	−0.7100	5.7520	/
−0.8586	10.9352	1.5034	−1.7881	0.7376	1.1129	/
−0.9376	0.3819	4.8547	−4.6069	1.0109	−6.2961	/

*Note. W*_1_ and *B*_1_ are the weights between the input and hidden layers, and *W*_2_ and *B*_2_ are the weights between the hidden and output layers for the trained GA-BPNN model.

## Supplementary Materials

Supplementary Materials To read our article more intuitively, the supplementary material of graphical abstract is added in Fig. S1.
