# Supplementary figures and images for: Optimization of Bioactive Ingredient Extraction from Chinese Herbal Medicine Glycyrrhiza glabra: A Comparative Study of Three Optimization Models
Source: Evid Based Complement Alternat Med. 2018 May 15;2018:6391414. doi: 10.1155/2018/6391414 (PMC5977065; doi:10.1155/2018/6391414)

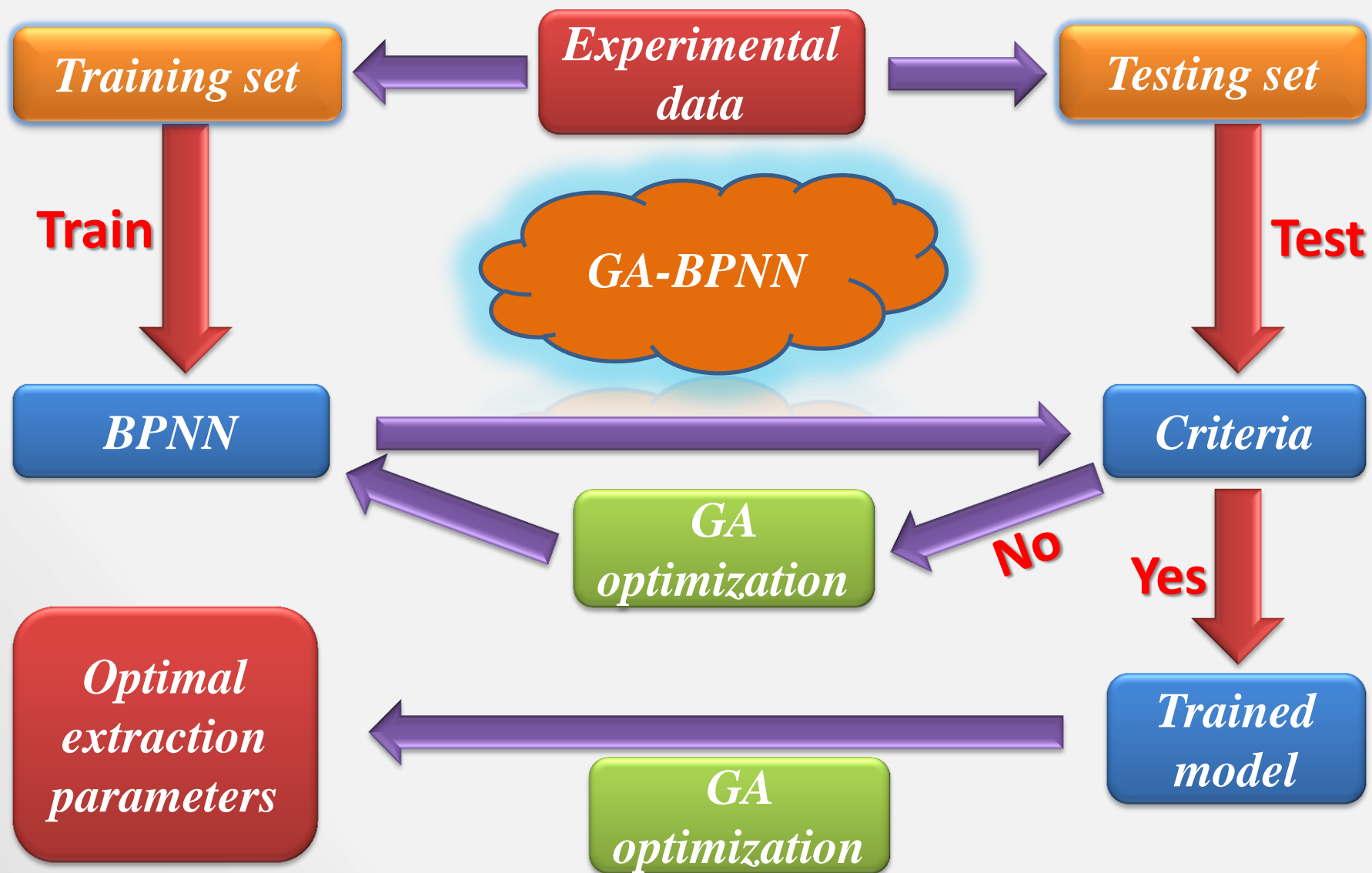

Supplement: Supplementary Materials — To read our article more intuitively, the supplementary material of graphical abstract is added in Fig. S1. [file 6391414.f1.pdf]
